# Understanding Practice Through Research

**DOI:** 10.1093/geroni/igaa057.2323

**Published:** 2020-12-16

**Authors:** Sara Stratton, Pi-Ju (Marian) Liu, Kendon Conrad, Zachary Hass

**Affiliations:** 1 San Francisco Adult Protective Services, San Francisco, California, United States; 2 Purdue University, West Lafayette, Indiana, United States; 3 University of Illinois at Chicago, Chicago, Illinois, United States

## Abstract

Substantiation of abuse, neglect, and exploitation (ANE) data collected during the Identification, Services, and Outcomes (ISO) Matrix pilot included some outlier scores warranting further investigation. To examine outliers and other unexpected findings in greater detail, we completed case reviews and caseworker interviews to explore why outlier cases did not follow the expected patterns of substantiation decisions. Cases were selected on a random basis from those with self-neglect outlier scores. Several factors were found to have contributed to the extreme scores in all three categories of confirmed, inconclusive, and unfounded abuse. In this study, no valid ISO Matrix scores were found that contradicted the substantiation decision. Instead, analysis revealed that most of the errors occurred: 1) in application of the intended procedures showing need for further training, and 2) some needed changes to the procedures of the ISO Matrix based on feedback from caseworkers.

